# Nutri-Score of Meat, Fish, and Dairy Alternatives: A Comparison between the Old and New Algorithm

**DOI:** 10.3390/nu16060892

**Published:** 2024-03-19

**Authors:** Sylvie Huybers, Annet J. C. Roodenburg

**Affiliations:** Department of Food & Industry, HAS Green Academy, Spoorstraat 62, 5911 KJ Venlo, The Netherlands; s.huybers@has.nl

**Keywords:** Nutri-Score, front-of-pack label (FOPL), meat alternatives, dairy alternatives, milk alternatives, plant-based, nutritional quality

## Abstract

Nutri-Score is a front-of-pack label that visualizes the nutritional quality of food products from most healthy (A, dark green) to least healthy (E, red). However, concerns have been raised about discrepancies between Nutri-Score labels and dietary recommendations. Therefore, the Nutri-Score algorithm has recently been adapted. To investigate the effect of the new algorithm, the Nutri-Score of plant-based meat, fish, and dairy alternatives (*n* = 916) was calculated with the old and new algorithms. In addition, the nutritional values of meat and milk alternatives with Nutri-Score labels A and B were compared under the old and new conditions and subsequently assessed for alignment with the criteria of Dutch dietary guidelines. The new algorithm resulted in a reduction in the number of products with labels A and B, ranging from 5% (cold cuts alternatives) to 55% (milk alternatives). The nutritional composition of products with labels A and B improved for meat alternatives (lower energy and saturated fatty acid contents; higher protein content) and milk alternatives (lower energy, salt, and sugar contents; higher protein and fiber contents). Overall, the new Nutri-Score algorithm is more in line with the Dutch dietary guidelines for plant-based meat and dairy alternatives, though challenges remain with respect to micronutrient (iron, calcium, vitamin B12), salt, and protein contents.

## 1. Introduction

Due to the increase in obesity and the related non-communicable diseases worldwide [1], the focus on a healthy diet has increased. Moreover, the role of a sustainable diet that has less impact on our climate has gained importance. Different initiatives, such as the EAT-Lancet Commission on Food, Planet, and Health, study the effect of our food choices on planetary boundaries [2]. Part of the EAT-Lancet diet entails the transition from a diet that mainly supplies animal-based proteins to a diet high in plant-based proteins. This change results in relevant beneficial effects on our climate [2]. The daily consumption of animal-based products is generally high in developed countries [3]. Currently, the Dutch population consumes 59% animal-based and 41% plant-based proteins [4], and the ambition is to reach a ratio of 50:50 of animal-based versus plant-based proteins, as described in the National Protein Strategy [5].

Next to sustainability, several studies have shown the positive effects of consuming a more plant-based (vegetarian) diet on health. It is correlated with a lower risk of cardiovascular diseases and certain types of cancer [6,7,8]. A fully plant-based diet is also linked to an improvement in weight management and body composition [9,10,11]. A higher satiety level after a meal and effects on gut microbiota composition are possible mechanisms that could lead to lower daily energy intake [12,13]. Therefore, a more plant-based diet is recommended in many national dietary guidelines [14].

In parallel, the number of available plant-based meat, fish, and dairy alternatives in the supermarkets has increased over the years [15]. This aids the consumer in the transition towards a more sustainable plant-based diet. Indeed, more people define themselves as flexitarian, which is defined as a person who consciously lowers their meat consumption by choosing to refrain from eating meat for several days a week. On average, one third of Europeans describe themselves as flexitarian [16], leading to increased sales of plant-based products [15]. This highlights the growing importance of this specific food category. However, the nutritional quality of plant-based meat and dairy alternatives is under discussion. The most frequently reported nutritional issues for meat alternatives are a high salt content and a low protein and iron content, whereas low protein and calcium levels are reported for dairy alternatives [17,18,19,20]. This could impair the consumption of a sustainable plant-based diet that is also healthy.

To promote healthy food choices, a large range of healthy products that can be easily distinguished as being healthy by the consumer should be available. To this end, different front-of-pack labels (FOPLs) are used, such as the Multiple Traffic Light, Key Hole, and Nutri-Score labels [21]. The results of the impact of FOPLs on food purchases vary among studies and depend on different factors, including the level of understanding of FOPLs, economic status, food category, and price [21].

The Nutri-Score is a FOPL that was established by France [22] and has been adopted by seven European countries as the preferred FOPL. It has been (voluntarily) implemented by these countries, including the Netherlands. It is classified as a summary FOPL that informs the consumer about the nutritional quality of a product by assigning points to positive aspects (protein, fiber, and fruit and vegetables content) and negative aspects (sugar, saturated fatty acids, salt, and energy content), which are summarized in a FOPL by combining letters (A–E) and colors (green–red) for the highest (A, dark green) and lowest (E, red) nutritional qualities. Different studies have shown a small effect of the Nutri-Score on healthy food purchases [23,24,25,26].

However, Nutri-Score labelling has also raised concerns about misleading information due to its discrepancy with national dietary guidelines [27,28]. For instance, our previous study, which assessed the healthiness of plant-based meat and dairy alternatives, revealed nutritional shortcomings (i.e., low protein, iron, and calcium contents) for products with healthy Nutri-Score labels A and B based on the criteria of Dutch dietary guidelines [29]. This is confusing for the consumer and could impair the effect of the FOPL on healthy product choices. Because of these concerns, an international scientific committee with representatives from the seven European countries that implemented this system has worked on a revision of the algorithm. This updated algorithm, with the aim of improving the nutritional profile of Nutri-Score labels, has been published for foods and beverages separately [22].

To investigate whether the new Nutri-Score algorithm is, indeed, an improvement on the old algorithm, this study evaluated the previously collected meat, fish, and dairy alternatives with both the old and new algorithm. In addition, for the food categories of meat alternatives and milk alternatives, the nutritional values were assessed for products with Nutri-Score labels A and B. This was carried out in order to explore whether the new algorithm is more in line with the criteria of the Dutch dietary guidelines for these food categories.

## 2. Materials and Methods

### 2.1. Product Selection and Data Collection

A total of 916 meat, fish, and dairy alternatives were selected from eight Dutch supermarkets in the period of March–May 2021, as previously described [29]. In short, the products were chosen using a few search terms (vegetarian, plant-based, meat and/or fish alternative, milk and/or cheese alternative, protein drink) in the webshop of the supermarkets. All the available products that belonged to one of the two categories—(1) ready-made meat, fish, and cold cuts alternatives and (2) plant-based protein drinks, desserts, and cheeses—were selected. The nutritional composition (energy, protein, total sugar, saturated fatty acid, salt, calcium, iron, and vitamin B12 as expressed per 100 g) and the product ingredients were obtained from the nutritional tables and ingredient lists, as indicated in the webshop. Online product information was not available for two of the supermarkets. The products (*n* = 42) that had not been included in the product database after the aforementioned online selection process were added via a physical visit to these two supermarkets. The protein energy % (E%) was calculated as the amount of energy from proteins (i.e., protein per 100 g × 4 Kcal) divided by the total energy (i.e., Kcal per 100 g) × 100. In the case of a nutrient not being mentioned in the nutritional table, the available amount of said nutrient in the product was assumed to be zero.

### 2.2. Nutri-Score Calculation

The Nutri-Score of the products was calculated according to the guidelines of Santé publique France [22] with both the old and the new—adapted in 2022 (for general foods) or 2023 (for beverages)—algorithms. The Nutri-Score divides food products into five categories based on their nutritional quality by combining letters (A to E) and colors. It ranges from a dark green A score (representing the highest nutritional quality) to a red E score (representing the lowest nutritional quality) (Table 1). The Nutri-Score algorithm assigns points based on nutrient content in 100 g of food or 100 mL of beverage. Positive points are obtained for nutrients or ingredients of which an adequate intake is considered healthy, i.e., the percentage of fruits, vegetables, pulses, nuts, and rapeseed, walnut, and olive oils (old algorithm) or the percentage of fruits, vegetables, and legumes (new algorithm), and the amount of fiber and protein. The fruits and vegetables component was calculated by the sum of each individual component (expressed as a % in the ingredient list). Negative points are attributed to each nutrient of which an excessive intake is considered unhealthy, i.e., energy density and the amount of sugars, saturated fatty acids, and salt. The total points, also called the FSAm-NPS score, are calculated by subtracting the sum of positive points from the sum of negative points. The final Nutri-Score is based on the cutoff points indicated in Table 1. All the products were analyzed according to the algorithm for general foods, except for plant-based protein drinks, which were calculated as general foods in the old algorithm and as beverages in the new algorithm. The Nutri-Score was based on the individual calculations and not on the Nutri-Score presented on the package, if applicable.

### 2.3. Data Analysis

Data were collected in Excel (Microsoft 365) and analyzed using the SPSS statistical software (IBM, Chicago, IL, USA, version 28). The FSAm-NPS score was analyzed as a continuous variable. The normal distribution of the FSAm-NPS score and the nutritional values was evaluated with a Shapiro–Wilk test. The values were not normally distributed and are, therefore, expressed as a median (25–75 percentile). To assess whether the FSAm-NPS score is different between the old and new Nutri-Score algorithms for each food category, the Wilcoxon Signed Rank test for dependent samples was used. The Mann–Whitney U test for independent samples was used to assess differences in the nutritional values of products that fell into either the A or B Nutri-Score label, as analyzed with the old and new algorithms. A *p*-value < 0.05 was considered statistically significant.

## 3. Results

### 3.1. Nutri-Score for Meat, Fish, and Cold Cuts Alternatives

**Figure 1:**
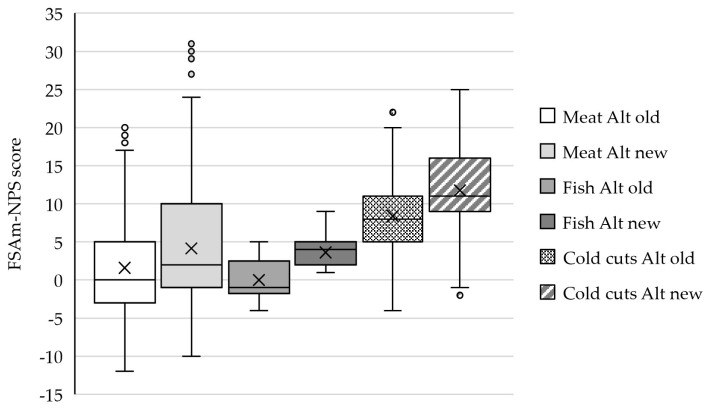
FSAm-NPS score of plant-based meat (*n* = 445), fish (*n* = 16), and cold cuts (*n* = 59) alternatives according to the old and new Nutri-Score algorithms. Data are presented as the median, with 25 and 75 percentiles. X = mean value; and Alt = alternatives.

**Figure 2:**
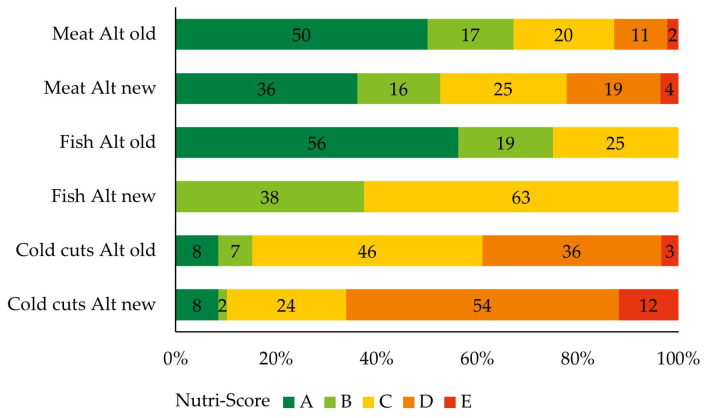
Nutri-Score label distribution of meat (*n* = 445), fish (*n* = 16), and cold cuts (*n* = 59) alternatives according to the old and new Nutri-Score algorithms. Data are presented as the % of the total products per category. Alt = alternatives.

Figure 1 shows the FSAm-NPS scores as analyzed with the old and new Nutri-Score algorithms for each food category. The median FSAm-NPS score significantly increased with the new algorithm for plant-based meat, fish, and cold cuts alternatives, scoring, respectively, 2, 5, and 3 points. This resulted in a shift towards a Nutri-Score label indicating a lower nutritional quality (Figure 2). The percentage of products with a healthy label A or B decreased with 5% for cold cuts alternatives, 15% for meat alternatives, and 37% for fish alternatives. With the new algorithm, none of the fish alternatives received a Nutri-Score label A.

For the group of plant-based meat alternatives with a healthy Nutri-Score label A or B, the nutritional values of each label were compared between the old and new algorithms (Table 2). The protein and energy content of the products with labels A and B significantly improved with the new algorithm. The protein content was complying with the Dutch dietary criterium level (>20 E%) in 88% and 75% of the products with the new Nutri-Score labels A and B, respectively. For the products with label B, the amount of saturated fatty acids was also significantly reduced (25%) with the new algorithm. When compared to the criteria of the Dutch dietary guidelines for plant-based meat alternatives, the majority of products with labels A and B did not meet the required salt, iron, and vitamin B12 levels.

### 3.2. Nutri-Score for Milk, Dessert, and Cheese Alternatives

**Figure 3:**
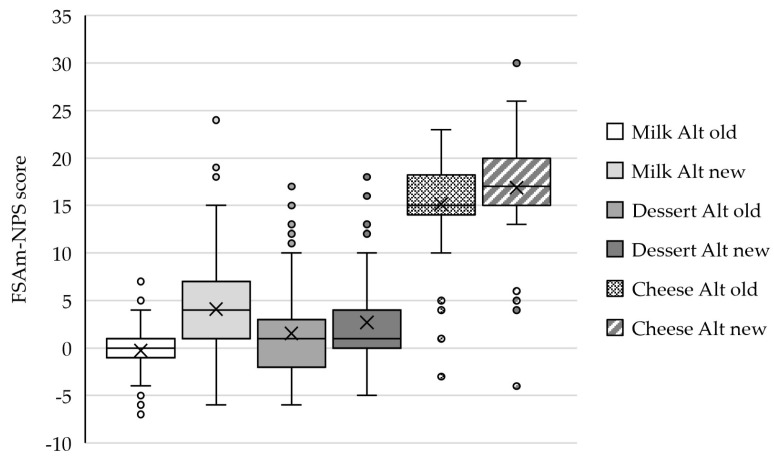
FSAm-NPS score of plant-based milk (*n* = 211), dessert (*n* = 127), and cheese (*n* = 58) alternatives according to the old and new Nutri-Score algorithms. Data are presented as the median, with 25 and 75 percentiles. X = mean value; and Alt = alternatives.

The median FSAm-NPS scores, as calculated with the new Nutri-Score algorithm, significantly increased, with, respectively, 4 and 2 points for milk and cheese alternatives (Figure 3). The median score for dessert alternatives did not change, though the 25–75 percentile distribution significantly shifted towards 0–4 compared to the old algorithm (−2–3).

**Figure 4:**
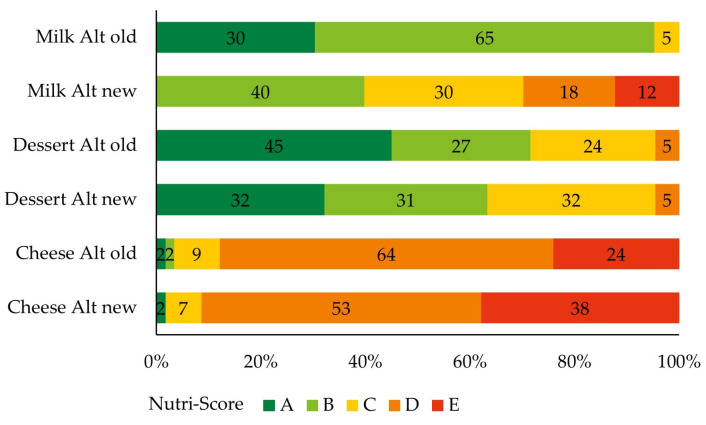
Nutri-Score label distribution of milk (*n* = 211), dessert (*n* = 127), and cheese (*n* = 58) alternatives according to the old and new Nutri-Score algorithms. Data are presented as the % of the total products per category. Alt = alternatives.

Figure 4 demonstrates the resulting Nutri-Score labels. The number of products with labels A or B showed a minor decline for dessert (10%) and cheese (2%) alternatives. For the category of milk alternatives, the amount of products with an A or B label reduced from 95% with the old algorithm to 40% with the new algorithm. None of the plant-based milk alternatives received label A with the new algorithm.

Consequently, the nutritional values of milk alternatives could only be compared between the old and new algorithms for Nutri-Score label B (Table 3). For this label, the protein, fiber, energy, sugar, and salt contents were more favorable according to the new algorithm compared to the old algorithm (*p* < 0.05). However, the protein content of 55% of the products with label B still did not meet the criterium of ≥20 E% protein set by the Dutch dietary guidelines for healthy plant-based milk alternatives. In addition, the majority did not meet the minimal requirements for calcium and vitamin B12 supplementation.

## 4. Discussion

This study evaluated how the updated Nutri-Score algorithm classified plant-based meat, fish, and dairy alternatives compared to the old algorithm. For all the subcategories, less products were classified as being healthy (i.e., dark green label A or light green label B) with the updated Nutri-Score algorithm. The biggest changes were observed for plant-based milk alternatives.

The distribution of Nutri-Score labels, based on the old algorithm, for plant-based meat and dairy alternatives is comparable to the results found in other studies [18,30,31]. This indicates that a representative selection of plant-based products was made for the inventory in this study. To date, information about the effect of the updated Nutri-Score algorithm is scarce. However, all the available studies describe a decline in products with labels A or B and/or a better alignment with national dietary guidelines for most food subcategories [32,33,34,35,36,37]. The observed low percentage (40%) of plant-based milk alternatives with labels A and B according to the updated algorithm in the current study was supported by Øvebrø et al., who found 26% of milk alternatives belonging to the new Nutri-Score labels A and B [32]. In addition, Sarda et al. reported a 76% decline in ultra-processed plant-based milk alternatives with labels A and B [34].

This suggests that the new algorithm is more strict and better suited to discriminating healthier and unhealthier products. For plant-based meat, fish, dessert, and cheese alternatives belonging to the group of general foods, this can be explained by more negative points being attributed to a high salt content (20 instead of 10) and more positive points for products high in protein content (7 instead of 5), combined with the use of a broader nutrient range of salt, protein, and fiber contents (Table 1) [22]. Indeed, protein content significantly improved for meat alternatives with labels A and B in the updated algorithm, whereas the adaptations did not affect the overall salt content. The salt content remained above that recommended in the Dutch dietary guidelines in the majority of the products with label B. The applied broader range and the allocation of more maximum points to the salt content mainly affect products with a salt content above 2.2 g/100 g, whereas all the meat alternatives with labels A and B in this study are below this level. The possibility to weigh positive points against negative points in the Nutri-Score algorithm might stimulate the reformulation of products by the food industry to, e.g., further decrease salt content, and ultimately improve the Nutri-Score labels. The protein content meets the criteria for most of the plant-based meat alternatives with labels A or B, indicating that the new Nutri-Score algorithm is more suitable to distinguishing between protein-rich and protein-poor plant-based substitutes. Nevertheless, a small number of meat alternatives still have protein levels below the criteria levels of Dutch dietary guidelines.

Plant-based milk alternatives are calculated as beverages instead of general foods according to the updated Nutri-Score guidelines, according to which mainly energy and sugar content are more critically weighed and exclusively waters receive Nutri-Score label A. This clarifies the pronounced decline in products with labels A and B and the consequently higher protein and lower sugar contents for the products with label B. In our opinion, this is a major improvement since dairy products are one of the main contributors (24%) to daily protein intake for the Dutch population [38]. Of notice, 55% of milk alternatives with label B still did not meet the criteria of >20 E% protein in the updated algorithm. This indicates that the algorithm is improved for milk alternatives but not yet optimal to be in line with Dutch dietary recommendations [14].

Both for plant-based meat alternatives and milk alternatives with label B, the energy density decreased with the new algorithm. This is mainly related to a reduction in fat and sugar for meat alternatives and a total drop in sugar content for plant-based milk alternatives with label B. This could be favorable for daily energy intake and, in the long-term, for weight control. Since plant-based sources have generally a higher satiating effect [10,12], no compensation is expected for the lower calory density.

Despite improvements in the algorithm, the Nutri-Score does not take mineral (iron and/or calcium) and vitamin B12 content into account for meat and dairy alternatives. The majority of products with labels A and B do not contain any mineral or vitamin fortification. Notably, a product either contains the required amount of fortified minerals and or vitamins or none at all. This is brand-specific or relates to a product having an organic logo. For milk alternatives and other dairy products, this supplementation is essential for consumers on a full plant-based diet, since 57% of calcium intake in a non-plant-based diet comes from dairy products [38]. This also accounts for iron and vitamin B12 [38]. The optimal absorption of heme-iron from animal-based products [39] or limited options to fully compensate this with other natural plant-based sources make the fortification of these micronutrients relevant. Because it is not expected that this will be incorporated into the Nutri-Score algorithm, clear information on a product’s package and communication to the consumer about relevant mineral and vitamin contents remain essential to the making of healthy plant-based product choices. To fully mitigate these nutritional aspects, in our opinion, specific legislation is required for plant-based meat and dairy alternatives that have the intention to completely substitute their animal-based counterparts.

## 5. Conclusions

The updated Nutri-Score algorithm can better discriminate healthy plant-based meat, fish, and dairy alternatives mainly due to the algorithm adaptations that value protein content. However, the majority of plant-based meat and milk alternatives with Nutri-Score labels A and B are still not fully in line with Dutch dietary guidelines because of the high salt content in meat alternatives and insufficient mineral and vitamin fortification in both meat and milk alternatives. This remains a concern that needs to be addressed in order to facilitate healthy plant-based choices in the supermarket setting. We recommend a separate Nutri-Score food category for plant-based alternatives that are explicitly sold as full replacers of their animal-based counterparts, with an algorithm which also takes micronutrient composition into account. Otherwise, legislation is necessary to regulate the required front-of-pack information about micronutrient and protein contents to easily inform the consumer about which products are the healthiest plant-based meat, fish, and dairy alternatives.

## Figures and Tables

**Table 1:** The Nutri-Score front-of-pack logo and the main differences between the old and new Nutri-Score algorithms that are relevant for plant-based meat, fish, and dairy alternatives.

	Nutri-Score General							
Nutritional quality	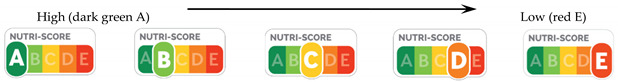							
	**Nutri-Score Old**				**Nutri-Score New**			
Product categories	General foods Added fats Cheeses Beverages				General foods Fats, oils, nuts, and seeds Cheeses Beverages Red meat			
	**General Foods Old**		**General Foods New**		**Beverages Old**		**Beverages New**	
Specific profiling characteristics	Include plant-based meat, fish, milk, dessert, and cheese Content of fruits, vegetables, legumes, nuts, and oils (rapeseed, walnut, and olive) in %		Include plant-based meat, fish, dessert, and cheese Content of fruits, vegetables, and legumes in %		Do not include plant-based milk Content of fruits, vegetables, legumes, nuts, and oils (rapeseed, walnut, and olive) in %		Include plant-based milk Content of fruits, vegetables, and legumes in %	
Range (min–max) of allocated points with the related nutritional values per component:	Points	Values/100 g	Points	Values/100 g	Points	Values/100 mL	Points	Values/100 mL
Energy (KJ)	0–10	≤335–≥3350	0–10	≤335–≥3350	0–10	0–≥270	0–10	≤30–≥390
SFA (g)	0–10	≤1–≥10	0–10	≤1–≥10	0–10	≤1–≥10	0–10	≤1–≥10
Sugar (g)	0–10	≤4.5–≥45	0–15	≤3.4–≥51	0–10	0–≥13.5	0–10	≤0.5–≥11
Salt (g)	0–10	≤0.225–≥2.25	0–20	≤0.2–≥4	0–10	≤0.225–≥2.25	0–20	≤0.2–≥4
Protein (g)	0–5	≤1.6–≥8.0	0–7	≤2.4–≥17	0–5	≤1.6–≥8.0	0–7	≤1.2–≥3
Fiber (g)	0–5	≤0.9–≥4.7	0–5	≤3.0–≥7.4	0–5	≤0.9–≥4.7	0–5	≤3.0–≥7.4
FVL (%) ^1^	0–5	≤40–≥80	0–5	≤40–≥80	0–5	≤40–≥80	0–6	≤40–≥80
NNS							0–4	absent–present
FSAm-NPS score:								
Nutri-Score A	Min to −1		Min to 0		Waters		Waters	
Nutri-Score B	0 to 2		1 to 2		Min to 1		Min to 2	
Nutri-Score C	3 to 10		3 to 10		2 to 5		3 to 6	
Nutri-Score D	11 to 18		11 to 18		6 to 9		7 to 9	
Nutri-Score E	19 to max		19 to max		10 to max		10 to max	

The score ranges from products with a high nutritional quality (A, dark green) to products with a low nutritional quality (E, red). The total (FSAm-NPS) score is based on the sum of positive points for nutrients with a positive effect on health (fiber, protein, % FVL) and negative points for nutrients with a negative effect on health (energy density, sugars, saturated fatty acids, salt). SFA = saturated fatty acids; FVL = fruits, vegetables, legumes; NNS = non-nutritive sweeteners; min = minimum; and max = maximum. ^1^ Includes nuts and rapeseed, walnut, and olive oils only in the old algorithm.

**Table 2:** Nutritional values of meat alternatives (*n* = 445) with Nutri-Score labels A or B according to the old and new algorithms, including the % of products which meet the criteria of Dutch dietary guidelines.

	Unit	Nutri-Score A Old (*n* = 223)	Nutri-Score A New (*n* = 161)	Nutri-Score B Old (*n* = 76)	Nutri-Score B New (*n* = 73)	Criteria
Protein	E%	32.7 (20.7–46.0)	41.3 (28.4–49.9) *	23.3 (13.7–33.9)	30.3 (20.4–41.2) *	≥20
	Meet criteria (%)	75	88	59	75	
Fiber	g/100 g	5.10 (3.80–6.40)	5.50 (3.25–6.55)	3.40 (1.85–4.28)	4.00 (1.80–5.68)	x
	Meet criteria (%)	x	x	x	x	
Energy	Kcal/100 g	175 (135–205)	165 (127–198) *	202 (176–241)	186 (168–219) *	x
	Meet criteria (%)	x	x	x	x	
Sugar	g/100 g	1.30 (0.60–2.80)	1.00 (0.50–2.25)	1.80 (1.00–2.8)	1.50 (0.70–2.45)	x
	Meet criteria (%)	x	x	x	x	
SFA	g/100 g	0.90 (0.60–1.30)	0.90 (0.60–1.20)	1.25 (0.90–2.00)	1.00 (0.75–1.55) *	≤2.5
	Meet criteria (%)	94	94	83	93	
Salt	g/100 g	1.10 (0.84–1.30)	1.00 (0.55–1.20)	1.30 (1.10–1.49)	1.30 (1.10–1.49)	≤1.125
	Meet criteria (%)	60	65	34	27	
Iron	mg/100 g	0.00 (0.00–2.10)	0.00 (0.00–2.10)	0.00 (0.00–2.10)	0.00 (0.00–2.10)	≥0.8
	Meet criteria (%)	33	27	32	44	
VitB12	mcg/100 g	0.00 (0.00–0.38)	0.00 (0.00–0.30)	0.00 (0.00–0.30)	0.00 (0.00–0.38)	≥0.24
	Meet criteria (%)	31	27	26	38	

Data are presented as the median (25–75 percentile). * *p* < 0.05 compared to the old algorithm of the same Nutri-Score label. SFA = saturated fatty acids. Criteria are according to the Dutch dietary guidelines for plant-based meat alternatives [14].

**Table 3:** Nutritional quality of plant-based milk alternatives (*n* = 211) with Nutri-Score labels A or B according to the old and new algorithms, including the % of products which meet the criteria of Dutch dietary guidelines.

	Unit	Nutri-Score A Old (*n* = 64)	Nutri-Score A New (*n* = 0)	Nutri-Score B Old (*n* = 137)	Nutri-Score B New (*n* = 84)	Criteria
Protein	E%	5.18 (27.60–36.04)	x	4.94 (2.34–10.29)	15.38 (5.00–31.58) *	≥20
	Meet criteria (%)	59		3	45	
Fiber	g/100 g	1.05 (0.50–3.85)	x	0.30 (0.10–0.60)	0.50 (0.13–1.38) *	x
	Meet criteria (%)	x		x	x	
Energy	Kcal/100 g	41.0 (35.3–52.0)	x	45.0 (26.5–53.0)	34.0 (24.0–46.5) *	x
	Meet criteria (%)	x		x	x	
Sugar	g/100 g	0.60 (0.00–2.45)	x	3.80 (0.30–5.50)	0.00 (0.00–0.80) *	≤6
	Meet criteria (%)	98		80	100	
SFA	g/100 g	0.30 (0.20–0.40)	x	0.20 (0.10–0.40)	0.30 (0.20–0.40)	≤1.1
	Meet criteria (%)	100		91	96	
Salt	g/100 g	0.09 (0.00–0.11)	x	0.10 (0.08–0.13)	0.09 (0.03–0.11) *	≤0.15
	Meet criteria (%)	92		96	98	
Ca	mg/100 g	0.00 (0.00–120)	x	0.00 (0.00–120)	0.00 (0.00–120)	≥80
	Meet criteria (%)	41		34	39	
VitB12	mcg/100 g	0.00 (0.00–0.38)	x	0.00 (0.00–0.35)	0.00 (0.00–0.38)	≥0.24
	Meet criteria (%)	39		28	37	

Data are presented as the median (25–75 percentile). * *p* < 0.05 compared to the old algorithm of the same Nutri-Score label. SFA = saturated fatty acids; and Ca = calcium. Criteria are according to the Dutch dietary guidelines for plant-based milk alternatives [14].

## Data Availability

The datasets generated for this study are available upon request from the corresponding author due to ethical restrictions.
